# Histopathological results of radical prostatectomy specimen of men younger than 50 years of age at the time of surgery: possible implications for prostate cancer screening programs?

**DOI:** 10.1007/s00345-023-04287-1

**Published:** 2023-01-19

**Authors:** Gisa Mehring, Derya Tilki, Hans Heinzer, Thomas Steuber, Randi M. Pose, Imke Thederan, Lars Budäus, Georg Salomon, Alexander Haese, Uwe Michl, Tobias Maurer, Hartwig Huland, Markus Graefen, Hendrik Isbarn

**Affiliations:** 1 Martini-Clinic, Prostate Cancer Center Hamburg-Eppendorf, Martinistr. 52, 20246 Hamburg, Germany; 2 Department of Urology, University Hospital Hamburg-Eppendorf, Hamburg, Germany

**Keywords:** Prostate cancer, Radical prostatectomy

## Abstract

**Introduction:**

Prostate cancer (PCa) detection is usually achieved by PSA measurement and, if indicated, further diagnostics. The recent EAU guidelines recommend a first PSA test at the age of 50 years, if no family history of PCa or BRCA2 mutation exists. However, some men might harbor significant PCa at younger age; thus we evaluated the histopathological results of men treated with radical prostatectomy (RP) in their 40 s at our institution.

**Materials and methods:**

We relied on the data of all patients who underwent RP in our institution between 1992 and 2020 and were younger than 50 years at the time of surgery. The histopathological results are descriptively presented. Moreover, we tested the effect of a positive family history on the descriptive results.

**Results:**

Overall, 1225 patients younger than 50 years underwent RP at our institution. Median age was 47 years. Most patients showed favorable histopathological characteristics. However, 20% of patients had extraprostatic disease (≥ pT3a), 15% had ISUP Gleason grade group ≥ 3, and 7% had positive lymph nodes (pN1). Patients with a known positive family history did not have a higher rate of adverse disease as their counterparts with a negative family history.

**Discussion:**

Our data show that the majority of patients who were diagnosed with PCa at a very young age had favorable histopathological RP characteristics. However, a non-negligible proportion of patients already showed locally advanced disease and would have probably benefited from earlier PCa detection. This should be kept in mind when PCa screening recommendations are proposed.

## Introduction

Determination of the serum prostate-specific antigen (PSA) is currently the most frequently initially used tool for detection of prostate cancer (PCa). Despite its known limitations, PSA screening can potentially reduce one’s individual risk of PCa death up to approximately 40% [1–3]. However, one of PSA’s well-recognized shortcomings is its inability to reliably differentiate between suspected high-grade and low-grade PCa [4]. In consequence, clinicians commonly encounter elderly men and increased PSA levels. Diagnosis and possibly active treatment of rather non-life-threatening PCa might be a consequence. To overcome those shortcomings, PSA measurement in younger men might identify those at high risk of already harboring or subsequently developing significant PCa who need a thorough follow-up, while likewise identifying those at low risk of significant PCa [5].

The optimal age at which to start with first PSA testing is, however, not exactly defined. In the current EAU guidelines, PSA testing is recommended at the age of 50 years. In case of a positive PCa family history or men of African descent, the first PSA testing should start at 45 years and in case of a known BRCA2 mutation at the age of 40 years [6]. Since family history or BRCA mutation status is, however, not always known to the patients, it is possible that for some patients a first PSA testing at the age of 50 years might be associated with adverse oncological outcome. To better quantify this proportion of men, we assessed the number of patients and their histopathological profile who underwent radical prostatectomy (RP) at our department during almost the last three decades.

## Materials and methods

Between 1992 and 2020, overall *N* = 34,986 patients underwent RP at our institution. RPs were performed as an open retropubic RP or robotic-assisted RP (RARP). Of those patients, *N* = 1225 (3.5%) were at the time of RP 49 years or younger and formed our dataset. Of those, *N* = 376 men (30.3%) underwent RARP, while *N* = 849 men (69.3%) were treated with open RP. The choice of the surgical approach was generally made by patients’ and surgeons’ preferences. However, in the first few years after RARP implementation in our institution (which was in 2005), locally advanced/high-grade PCa was more commonly treated with the open surgical approach. Clinical parameters of those patients were retrospectively analyzed from a prospectively collected dataset. Variables of interest consisted of age at surgery (years), PSA value before surgery (ng/ml), biopsy ISUP Gleason grade group (1–5), pT stage (pT2 vs. pT3a vs. pT3b/pT4), pN stage (pN0 vs. pN1 vs. pNx), RP ISUP Gleason grade group (1–5), nerve-sparing surgery (unilateral vs. bilateral vs. none), surgical margin status (positive vs. negative), and family history of PCa (yes vs. no vs. unknown). Since only very few patients had pT4 disease (*N* = 6), those patients were included in the pT3b group. We furthermore evaluated the overall rate of adverse disease, which was defined as ≥ pT3a, ISUP RP Gleason grade group 4 or 5, or pN1 disease. The variables of interest were descriptively analyzed.

Secondly, we tested the impact of a positive family history on histopathological parameters (pT stage, pN stage, and ISUP RP-Gleason grade group). Patients with unknown family history status of PCa were excluded from this analysis. Family history was considered positive if a male relative (grandfather, father, uncle, or brother) was previously diagnosed with PCa. Evaluation of family history is a routine part of our upfront evaluation before RP since the early 2010s as thus not available for all patients of the current report.

Proportions were compared with the Chi-square test. A *p* value of < 0.05 indicated statistical significance. All analyses were performed with SPSS, Version 20.

## Results

The descriptive characteristics of the studied patient population is given in Table 1. Median age at surgery was 47 years. Median PSA at the time of RP was 5.9 ng/ml (IQR 4.3–8.9). The majority of patients had an ISUP biopsy Gleason grade group of 1 or 2 (*N* = 1001, 81.7%), while 8.3% (*N* = 102), 5.7% (*N* = 70), and 3.1% (*N* = 38) had ISUP biopsy Gleason grade group 3, 4, and 5, respectively. Most patients (*N* = 956, 78%) had organ-confined disease at final histology, while 12.5% (*N* = 153) and 8.5% (*N* = 104) showed a pT3a and pT3b/pT4 disease, respectively. The rate of lymph node metastasis was 7.3% (*N* = 90), and the overall rate of adverse pathology (defined as pT stage pT3a or higher, and/or ISUP Gleason grade group 4 or 5, and/or pN1 disease) was 22.3%.

**Table 1:** Patient descriptive of *N* = 1225 patients 49 years or younger at the time of surgery treated with radical prostatectomy between 1992 and 2020

Variables	
Age (years)	
Mean (median)	47 (47)
IQR	45–48
PSA (ng/mL)	
Mean (median)	8.7 (5.9)
IQR	4.3–8.9
Biopsy Gleason grade group	
1	587 (47.9%)
2	414 (33.8%)
3	102 (8.3%)
4	70 (5.7%)
5	38 (3.1%)
Missing	14 (1.1%)
pT stage	
pT2	956 (78%)
pT3a	153 (12.5%)
pT3b/pT4	104 (8.5%)
Missing	12 (1%)
pN stage	
pN0	810 (66.1%)
pN1	90 (7.3%)
pNx	311 (25.4%
Missing	14 (1.1%)
RP Gleason grade group	
1	295 (24.1%)
2	736 (60.1%)
3	129 (10.5%)
4	2 (0.2%)
5	51 (4.2%)
Missing	12 (1%)
Nerve-sparing surgery	
Bilateral	938 (76.6%)
Unilateral	181 (14.8%)
None	68 (5.6%)
Missing	38 (3.1%)
Surgical margin status	
Negative	1038 (84.7%)
Positive	177 (14.4%
Missing	10 (0.8%)
Rate of adverse pathology^a^	
Yes	271/1215 (22.3%)
Family history of prostate cancer	
Yes	233 (19.2%)
No	360 (29.7%)
Unknown	619 (51.1%)

^a^Defined as pT stage pT3a or higher and/or pN1 disease

When the respective surgical approaches (open RP vs. RARP) were compared, the following was observed: the mean number of lymph nodes removed was 13 in patients who underwent open RP and was 15 in patients treated with RARP (*p* = 0.03). No significant differences were noted with respect to pT stage or positive surgical margin rates (both *p* > 0.05). However, significant differences were observed with respect to the rates of lymph node metastases and ISUP Gleason grade group 5. Patients who underwent open RP had higher rates of lymph node metastases (8.4 vs. 6.2%; *p* < 0.001) and had higher rates of ISUP Gleason grade 5 (4.9 vs. 2.7%; *p* < 0.001).

**Fig. 1:**
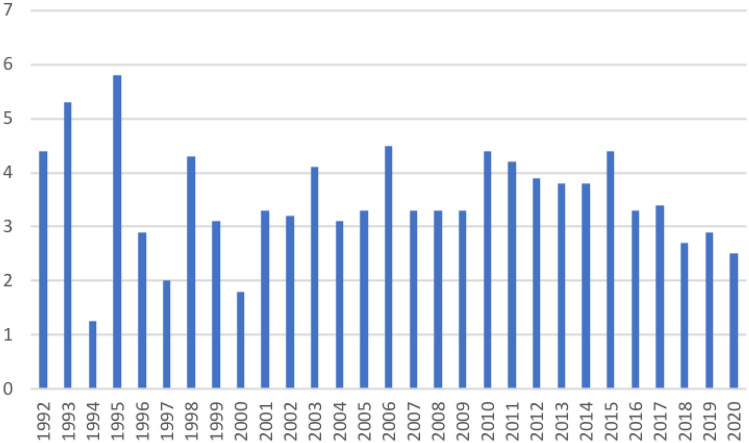
Proportion of patients treated with radical prostatectomy between 1992 and 2020, 49 years or younger at surgery. The *Y*-axis represents the proportion of men in percent in relation to all patients who underwent radical prostatectomy. The *X*-axis refers to the respective year of surgery

Figure 1 shows the yearly proportion of patients 49 years or younger at the time of surgery in relation to the entire cohort of patients treated with RP at our institution. It ranged from 1.25 to 5.8% (mean 3.2%). A clear increase or decrease over time was not observed.

Considering family history, overall 19.2% (*N* = 233) of the patients had one or more male relatives (grandfather, father, uncle, or brother) previously diagnosed with PCa. Conversely, 360 patients had a negative family history of PCa. In the remaining patients, family history was unknown. Due to the overall relatively small number of patients with a positive family history, no further differentiation of the family status (e.g., number of positive family members) was conducted. Table 2 shows the pT stage, pN stage, and ISUP RP Gleason grade group comparison between patients with a positive family history vs. those with a negative family history. Regarding pT stage, patients with a positive family history had lower rates of extraprostatic extension or seminal vesicle invasion (*p* = 0.001). Similarly, the rate of lymph node invasion (5.2 vs. 13.1%; *p* = 0.007) was significantly lower in patients with a positive family history. Finally, patients with a positive family history had lower rates of poorly differentiated tumor at final histology (ISUP Gleason grade 5). However, this difference failed to reach statistical significance (*p* = 0.12).

**Table 2:** Radical prostatectomy histopathological findings in patients with vs. without a positive family history of prostate cancer

Variables	Positive family history	Negative family history	*p* value
pT stage			
pT2	196 (84.1%)	251 (69.7%)	< 0.001
pT3a	22 (9.4%)	63 (17.5%)	
pT3b/pT4	15 (6.4%)	46 (12.7%)	
pN stage			
pN0	187 (80.3%)	261 (72.5%)	0.007
pN1	12 (5.2%)	47 (13.1%)	
pNx	34 (14.6%)	52 (14.4%)	
Radical prostatectomy ISUP Gleason grade group			
1	38 (16.3%)	59 (16.4%)	0.12
2	165 (70.8%)	225 (62.5%)	
3	19 (8.2%)	47 (13.1%)	
4	1 (0.4%)	1 (0.3%)	
5	10 (4.3%)	28 (7.8%)	

## Discussion

Even more than 30 years after the implementation of PSA testing into clinical practice, its optimal use for the detection of PCa is not clearly defined. One of the main criticism of PSA testing is that in daily routine, it is often used in elderly men. As the probability of PCa increases with advanced age, many men in their 60 s, 70 s, or even 80 s are diagnosed with PCa and subsequent active treatment with possible therapy-related side effects might occur. To reduce the probability of non-significant PCa detection, different diagnostic tools such as prostate MRI have been implemented into clinical routine within the last years. Prostate MRI has the potential to differentiate between significant and insignificant PCa in case of an elevated PSA [7] and to reduce the number of insignificant PCa diagnoses. Another approach to optimize PCa screening strategies is to take a first PSA test at a relative young age and to adopt further follow-up based on the amount of the PSA value. Such risk-adapted screening strategies are based on the observation that a baseline PSA test at young age can relatively precisely predict the risk of significant PCa development [5, 8].

Recently, the first results of the PROBASE study have been published [9]. The PROBASE study is a large randomized study with a sample size of more than 46,000 men, in which different risk-adapted PSA-screening strategies are explored. All men are 45 years old at study entry. One half of the patients are offered a PSA test at study entry and the other half a digital rectal examination only with a delayed first PSA test at the age of 50 years. The main aim of the study is to test the hypothesis that a delayed risk-adapted PSA screening starting at the age of 50 years is equally effective in preventing the rate of distant metastases between the two study arms, while reducing the rates of false positive PSA test (defined as a PSA value that gives rise to further diagnostics). This far, only the first results of the immediate PSA test group have been published. The number of biopsy-diagnosed PCa was 48 (0.2% of the entire screening arm). Of those, most men had an ISUP Gleason grade group of 1 or 2, while overall only four men had ISUP Gleason grade group 3, 4, or 5 disease. Those preliminary results are very encouraging. One has to wait for the results of the delayed PSA-screening group, though, to draw more robust conclusions from the trial.

In the current report, we assessed the number of patients who underwent RP at our institution and were younger than 50 years at the time of surgery. Moreover, the histopathological parameters of those patients were analyzed. Finally, a possible effect of PCa family history on histopathological outcomes was investigated. We found that the overall number of patients who were treated with RP at our institution during the last almost three decades was 1225, which was 3.5% of our RP patients. In total numbers, the mean number of young patients treated with RP at our clinic was 44 per year. Of those, the vast majority of patients had favorable disease characteristics, such as organ-confined disease and low ISUP Gleason grade pattern. However, 21% of the patients (*N* = 257) showed non-organ-confined disease at the final histopathological evaluation and 7% (*N* = 90) had evidence of lymph node metastases.

Given the long time span of the current evaluation (1992–2020), the overall number of patients with unfavorable disease characteristics appears rather low. However, one has to keep in mind that our institution (as every institution) is just one of many institutions in which RPs are performed. It is conceivable that our reported series is representative for other centers, as well. In consequence, the overall number of young patients with adverse PCa characteristics worldwide is surely non-negligible.

Our findings may impact PSA screening strategies. Although it is not exactly clear if earlier PCa detection would have resulted in better disease characteristics in all patients with advanced disease, it does not appear to be too farfetched if one states that some of the advanced disease stages could have been avoided by earlier PCa detection. Given that all patients of the current report were 49 years or younger at time of RP and some already showed advanced or poorly differentiated disease, it is likely that the prognosis of those men would have been even worse, if a first PSA would have been measured at the age of 50 years. Another aspect of interest is the functional outcome after definitive treatment. It is well known that functional outcomes, such as continence and potency rates after RP are usually better in younger individuals [10]. Regarding the optimal potency outcomes after RP, a bilateral nerve-sparing surgery is aspired. This is, however, only possible if the cancer is confined to the prostate gland. In consequence, one should pursue to detect significant PCa as early as possible to achieve optimal oncological and functional outcomes.

When the respective surgical approaches were compared (open RP vs. RARP), we found small, but statistically significant differences with respect to the number of lymph nodes removed in favor of the RARP approach. However, the mean number of removed lymph nodes was 13 vs. 15, which is probably of only little clinical relevance. Moreover, we found statistically significant differences between groups with respect to lymph node metastases and Gleason grade group 5 rates. This might be explained by the fact that within the first years after RARP implementation in our institution, locally advanced cases were predominantly operated with the open retropubic approach.

Interestingly, a positive family history was not associated with advanced disease in our series. This is in line with other previous publications [11, 12]. In fact, our findings showed quite the opposite: Men with a positive PCa family history had significantly lower rates of advanced pT stage and of lymph node metastases. Similar findings were observed when all patients (regardless of age) who underwent RP at our institution between 1992 and 2020 (*N* = 34,986) were evaluated (data not shown). The reason for this is not clear. A possible explanation might be that those patients are more aware of the disease in general and opt for earlier and more frequent PSA testing. However, this is just a hypothesis. Given that in a large number of patients of the current study, the family status was unknown, these results should be interpreted with caution. Nonetheless, our results show that positive family history was not associated with adverse histopathological results.

A limitation of the current study is its retrospective nature and the single center design. Moreover, our series did not include those patients who were younger than 50 years and already harbored advanced metastatic disease at diagnosis, since those patients are usually not candidates for RP. The true number of young patients with already advanced/metastatic PCa is thus unknown and surely higher than that reported in our series.

In summary, our data show that most patients younger than 50 years who were treated with RP at our institution over a time period of almost three decades had organ-confined disease. However, a small but non-negligible proportion of patients had aggressive and/or locally advanced disease. Family history of PCa does not appear to be associated with adverse pathology in young men. These findings should be kept in mind when discussing PSA-based screening strategies.
